# Supplementary material for: The importance of molecular characters when morphological variability hinders diagnosability: systematics of the moon jellyfish genus Aurelia (Cnidaria: Scyphozoa)
Source: PeerJ. 2021 Sep 9;9:e11954. doi: 10.7717/peerj.11954 (PMC8435205; doi:10.7717/peerj.11954)
Supplement: Supplemental Information 2 — Specimens are depicted in black, as vouchers, and features appear in red, as weighted averages of their contributions. Specimens highlighted in blue from the northeastern USA appear in Fig. 2 (YPM29380) and those highlighted in green from northeastern Canada and the southwestern USA in Fig. 5 (USNM30988 and USNM92912-5, respectively). One of the specimens highlighted in orange from the aquarium at Discovery Place, USA, appears in Fig. 6A (DP3-4). One of the specimens in the black boxes from the Arctic appears in Fig. 6C (USNM 44243-2) and the specimen from northwestern Canada in the grey box appears in Fig. 6D (USNM92913-1). Specimens highlighted in orange are Aurelia coerulea and in pink Aurelia cebimarensis sp. nov., identified based on genetic sequences (Table S4). See Table 1 for institution acronyms and Fig. 1, Tables S1-S3 for more information on specimens measured and morphological features. [file peerj-09-11954-s002.pdf]

Figure S2. Multidimensional scaling (MDS) of morphological features *without* estimation of missing data. Specimens are depicted in black, as vouchers, and features appear in red, as weighted averages of their contributions. Specimens highlighted in blue from the northeastern USA appear in Fig. 2 (YPM29380) and those highlighted in green from northeastern Canada and the southwestern USA in Fig. 5 (USNM30988 and USNM92912-5, respectively). One of the specimens highlighted in orange from the aquarium at Discovery Place, USA, appears in Fig. 6A (DP3-4). One of the specimens in the black boxes from the Arctic appears in Fig. 6C (USNM 44243-2) and the specimen from northwestern Canada in the grey box appears in Fig. 6D (USNM92913-1). Specimens highlighted in orange are *Aurelia coerulea* and in pink *Aurelia cebimarensis* sp. nov., identified based on genetic sequences (Table S4). See Table 1 for institution acronyms and Fig. 1, Tables S1-S3 for more information on specimens measured and morphological features.
