# Supplementary material for: The importance of molecular characters when morphological variability hinders diagnosability: systematics of the moon jellyfish genus Aurelia (Cnidaria: Scyphozoa)
Source: PeerJ. 2021 Sep 9;9:e11954. doi: 10.7717/peerj.11954 (PMC8435205; doi:10.7717/peerj.11954)
Supplement: Supplemental Information 3 — Colored circles represent bootstrap resampling frequencies and SH-aLRT (black = ≥95, grey = ≥75, absent = <75). When both of these values were not in the same range the lowest one was considered. Scale bar represents the number of nucleotide substitutions per site. Tables S4-S5 contains further details on sequences used to reconstruct this phylogeny and single-marker phylogenies are presented in Figs. S4-S7. [file peerj-09-11954-s003.pdf]

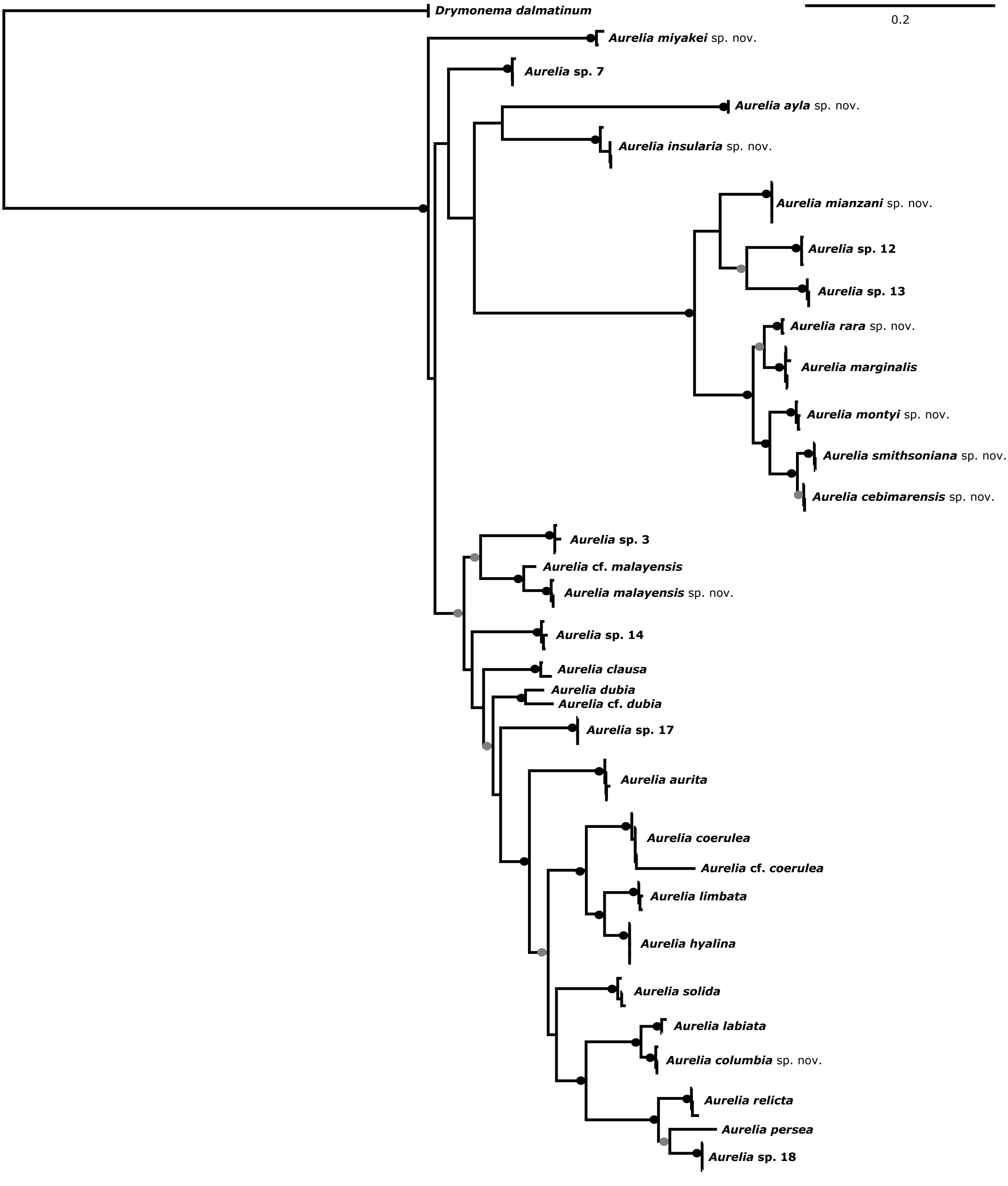

Figure S3. Concatenated phylogenetic analysis combining markers 16S, COI, ITS1 and 28S, reconstructing relationships between 28 *Aurelia* species hypotheses under maximum likelihood as the optimality criterion. Colored circles represent bootstrap resampling frequencies and SH-aLRT (black = ≥95, grey = ≥75, absent = <75). When both of these values were not in the same range the lowest one was considered. Scale bar represents the number of nucleotide substitutions per site. Tables S4-S5 contains further details on sequences used to reconstruct this phylogeny and single-marker phylogenies are presented in Figs. S4-S7.
