# Supplementary figures and images for: The importance of molecular characters when morphological variability hinders diagnosability: systematics of the moon jellyfish genus Aurelia (Cnidaria: Scyphozoa)
Source: PeerJ. 2021 Sep 9;9:e11954. doi: 10.7717/peerj.11954 (PMC8435205; doi:10.7717/peerj.11954)

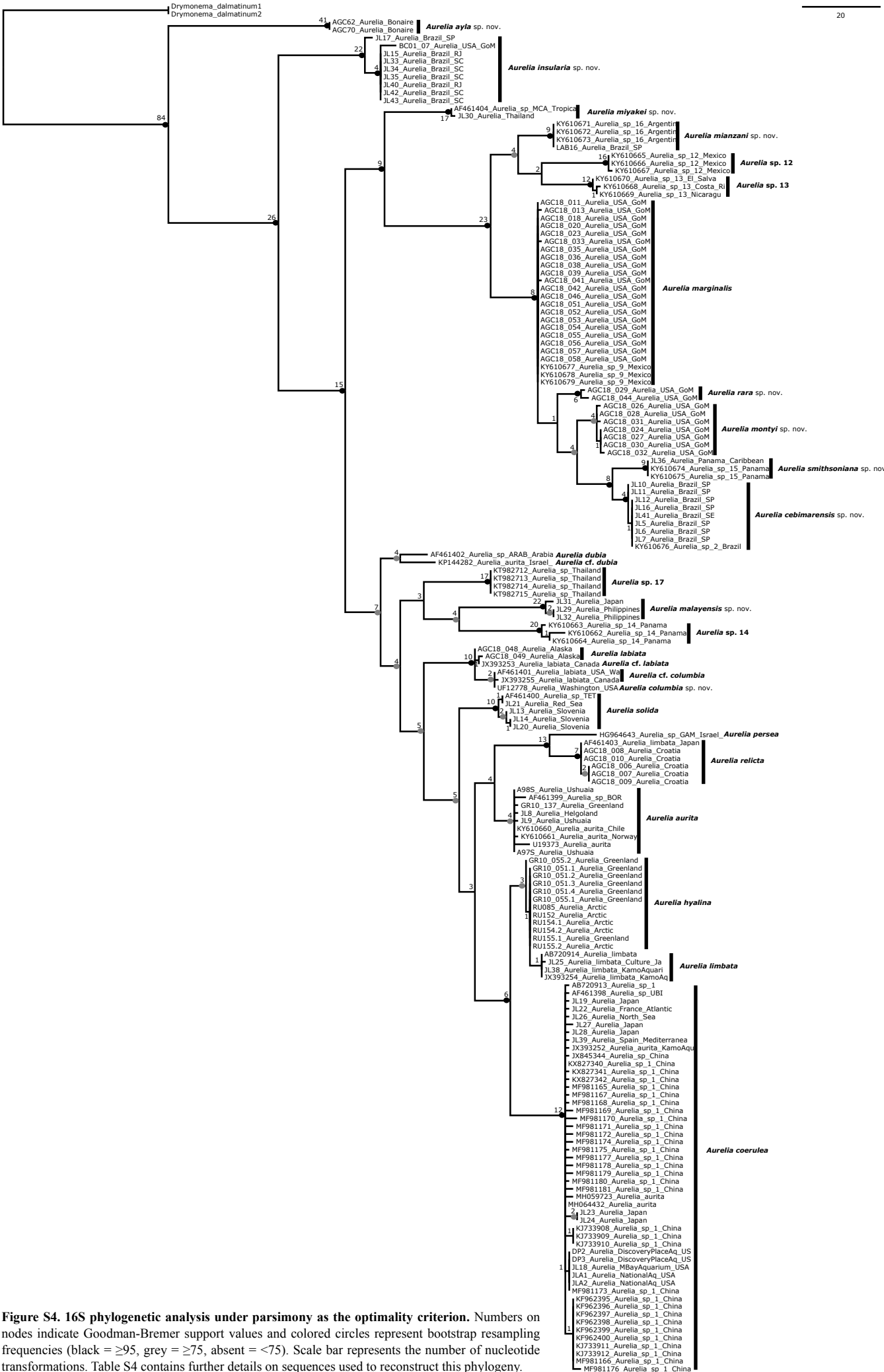

Supplement: Supplemental Information 4 — Numbers on nodes indicate Goodman-Bremer support values and colored circles represent bootstrap resampling frequencies (black = ≥95, grey = ≥75, absent = <75). Scale bar represents the number of nucleotide transformations. Table S4 contains further details on sequences used to reconstruct this phylogeny. [file peerj-09-11954-s004.pdf]
