# Supplementary material for: The importance of molecular characters when morphological variability hinders diagnosability: systematics of the moon jellyfish genus Aurelia (Cnidaria: Scyphozoa)
Source: PeerJ. 2021 Sep 9;9:e11954. doi: 10.7717/peerj.11954 (PMC8435205; doi:10.7717/peerj.11954)
Supplement: Supplemental Information 10 — Based on Dawson (2003), with some modifications (indicated by an “a”) and additions (f31-46 are either novel or from Gershwin, 2001 or Chiaverano et al., 2016). [file peerj-09-11954-s010.docx]

| **f1** | Bell diameter |
| --- | --- |
| **f2** | Manubrium depth |
| **f3a** | Number of oral arm folds (curving points per arm) |
| **f5** | Oral arm length |
| **f6** | Manubrium width |
| **f7** | Oral arm width (half-length) |
| **f8** | Gastric pouch shape: 1) u-shape, 2) horseshoe, 3) drop-like, 4) flat-u |
| **f9** | Proximal gastric diameter |
| **f10** | Distal gastric diameter |
| **f11a** | Lateral sub-genital pore diameter (furthest points) |
| **f12** | Sub-genital pore position: 1) central, 2) inside, 3) overlapping, 4) outside |
| **f13a** | Thickness of mesoglea surrounding sub-genital pore |
| **f19** | Number of lobes |
| **f20** | Number of rhopalia |
| **f21** | Bell shape determined by the best-fit curve through measurements of bell height: 1) concave, 2) convex, 3) flat |
| **f22a** | Bell thickness (center) |
| **f23** | Perradial origins (per quadrant) |
| **f24** | Interradial origins (per quadrant) |
| **f25a** | Adradial origins (per octant) |
| **f26** | Perradial anastomoses (per quadrant) |
| **f27** | Interradial anastomoses (per quadrant) |
| **f28a** | Adradial anastomoses (f37 + f38) (per octant) |
| **f29** | Rhopaliar indent |
| **f30** | Non-rhopaliar indent |
| **f31** | Size of gastric pouches (calculated by subtracting ½ of f9 from ½ of f10) |
| **f32** | Length of rhopalia measured from the base to the tip of rhopaliar lobe |
| **f33** | Maximum number of branching points of the canal system within a perradial quadrant |
| **f34** | Lateral gastric diameter (furthest points) |
| **f35** | Distance between gastric pouch proximal edge tips |
| **f36** | Distance between proximal edge tips of adjacent gastric pouches |
| **f37** | Perradial-adradial anastomoses (per quadrant) |
| **f38** | Interradial-adradial anastomoses (per quadrant) |
| **f39** | Perradial terminations (per quadrant) |
| **f40** | Interradial terminations (per quadrant) |
| **f41** | Adradial terminations (per octant) |
| **f42** | Perradial branching points (per quadrant) |
| **f43** | Interradial branching points (per quadrant) |
| **f44** | Adradial branching points (per octant) |
| **f45** | Shape of sense organ (direction of rhopaliar club and shape of its hood) |
| **f46** | Shape of manubrium |

**Table S3. Morphological features recorded per medusa.** Based on Dawson (2003), with some modifications (indicated by an “a”) and additions (f31-46 are either novel or from Gershwin, 2001 or Chiaverano *et al.*, 2016).
